# FEELING ALONE TOGETHER: LONELINESS IN OLDER ADULTS WITH COGNITIVE IMPAIRMENT AND THEIR CAREGIVERS WITH LOW MASTERY

**DOI:** 10.1093/geroni/igad104.1219

**Published:** 2023-12-21

**Authors:** Pildoo Sung, Jeremy Lim-Soh, Angelique Chan

**Affiliations:** Hong Kong Baptist University, Hong Kong, Hong Kong; Duke-NUS Medical School, Singapore, Singapore; Duke-NUS Medical School, Singapore, Singapore

## Abstract

Loneliness can be contagious because lonely people tend to share their loneliness with others. According to this perspective, loneliness in older persons with cognitive impairment (PCI) may beget loneliness in their family caregivers. However, not all caregivers of lonely PCI experience loneliness. This may be because caregivers have buffering resources that mitigate the contagion of loneliness. Nevertheless, empirical evidence on whether and how PCI and caregiver loneliness are related is lacking. Therefore, we examined the association between PCI loneliness and caregiver loneliness, and the moderation role of caregiver mastery on this association. We used dyadic data from 135 PCI and their family caregivers in Singapore. Descriptive statistics and bivariate correlation showed that PCI reported higher levels of loneliness than their caregivers, and PCI and caregiver loneliness were weakly related. Multivariable regression showed that PCI loneliness was not associated with caregiver loneliness, taking other covariates into account. Nevertheless, we found that the interaction between PCI loneliness and caregiver sense of mastery was significant, such that PCI loneliness was significantly associated with caregiver loneliness when caregivers had low mastery. In conclusion, lonely PCI may share their loneliness with their caregivers, and this may lead to caregiver loneliness if caregivers have low mastery. Tailored interventions should be designed to reduce loneliness among PCI and their caregivers and improve caregiver mastery as a protective factor against the spread of loneliness between PCI and caregivers.

